# The ERK and JNK pathways in the regulation of metabolic reprogramming

**DOI:** 10.1038/s41388-018-0582-8

**Published:** 2018-11-28

**Authors:** Salvatore Papa, Pui Man Choy, Concetta Bubici

**Affiliations:** 1 Cell Signaling and Cancer Laboratory, Leeds Institute of Cancer and Pathology, Faculty of Medicine and Health, University of Leeds, St James’ University Hospital, Beckett Street, Leeds, UK; 2 Department of Research & Development, hVIVO PLC, Biopark, Broadwater Road, Welwyn Garden City, UK; 3 College of Health and Life Sciences, Department of Life Sciences, Institute of Environment, Health and Societies, Division of Biosciences, Brunel University London, Uxbridge, UK; 4 Department of Medicine, Faculty of Medicine, Imperial College London, London, UK

**Keywords:** Cell signalling, Cancer metabolism

## Abstract

Most tumor cells reprogram their glucose metabolism as a result of mutations in oncogenes and tumor suppressors, leading to the constitutive activation of signaling pathways involved in cell growth. This metabolic reprogramming, known as aerobic glycolysis or the Warburg effect, allows tumor cells to sustain their fast proliferation and evade apoptosis. Interfering with oncogenic signaling pathways that regulate the Warburg effect in cancer cells has therefore become an attractive anticancer strategy. However, evidence for the occurrence of the Warburg effect in physiological processes has also been documented. As such, close consideration of which signaling pathways are beneficial targets and the effect of their inhibition on physiological processes are essential. The MAPK/ERK and MAPK/JNK pathways, crucial for normal cellular responses to extracellular stimuli, have recently emerged as key regulators of the Warburg effect during tumorigenesis and normal cellular functions. In this review, we summarize our current understanding of the roles of the ERK and JNK pathways in controlling the Warburg effect in cancer and discuss their implication in controlling this metabolic reprogramming in physiological processes and opportunities for targeting their downstream effectors for therapeutic purposes.

## Introduction

Cellular metabolism is the process by which a living cell converts nutrients into energy or new macromolecules through a series of biochemical reactions, known as catabolic pathways and anabolic pathways, respectively [1]. Through catabolic pathways, carbon fuels such as glucose, fatty acids, and glutamine are broken down to generate energy in the form of adenosine triphosphate (ATP), which is used to maintain cellular functions and construct new cellular components [1, 2]. There are two major ATP-producing pathways in mammalian cells, glycolysis and oxidative phosphorylation (OXPHOS) [3]. These two metabolic pathways function in concert to provide energy for cellular and tissue homeostasis. During glycolysis, a normal differentiated cell oxidizes glucose into pyruvate, which enters the mitochondria to be further oxidized to carbon dioxide in the tricarboxylic acid (TCA) cycle, producing the reduced electron carriers nicotinimide adenine dinucleotide (NADH) and flavin adenine dinucleotide (FADH2). NADH and FADH2 are then used during OXPHOS to generate 36 molecules of ATP per molecule of glucose. OXPHOS is the main ATP-producing pathway in normal cells and is strictly dependent on the presence of oxygen [3]. In fact, in the absence of oxygen, the pyruvate produced by glycolysis is converted to lactate with a net production of two molecules of NADH and only two molecules of ATP. Such a low ATP-producing pathway is referred to as anaerobic glycolysis or glucose fermentation and takes place in the cytoplasm. Thus, to survive in low oxygen conditions, a normal differentiated cell re-adjusts its glucose metabolism by shifting toward anaerobic glycolysis. Unexpectedly, rapidly proliferating tumor cells adopt this inefficient ATP-producing pathway as their chief manner of energy harvest even in the presence of sufficient levels of oxygen [4–6]. This is not a new concept as it was anticipated by Otto Warburg more than 90 years ago when he observed that tumor slices and ascites cancer cells display an enhanced rate of glycolysis and produce larger amount of lactate compared to their normal counterparts despite the presence of adequate levels of oxygen for mitochondrial respiration [7]. Subsequent works have revealed that this metabolic phenomenon is not restricted to cancer cells, but it is a common metabolic feature among all mammalian cells during periods of rapid proliferation [8–10].

Over the past decade, aerobic glycolysis has taken center stage in cancer research, because it is characteristic of essentially all types of cancer as well as its implications for cancer diagnosis and monitoring [4–6, 11–14]. It is now appreciated that aerobic glycolysis in cancer occurs downstream of molecular signaling pathways, often driven by mutations in oncogenes or tumor suppressors [15–18]. Evidence have pointed out that signaling pathways involving oncogenes and tumor suppressors play a direct role in promoting the conversion of energy metabolism to aerobic glycolysis in addition to their well-known functions in inducing aberrant cell proliferation or attenuating apoptosis [19–22]. Among the many signaling pathways that respond to oncogenic mutational events and regulate proliferation and apoptosis as well as aerobic glycolysis are members of the mitogen-activated protein kinases (MAPKs) family. There are three well-characterized subfamilies of MAPKs in mammals: the extracellular signal-regulated kinases (ERKs), the c-Jun N-terminal kinases (JNKs), and the p38 kinases [23]. Activation of each MAPK signaling follows a three-tier kinase module in which a MAP3K phosphorylates and activates a MAP2K, which in turn phosphorylates and activates a MAPK. Once activated, the MAPKs control a diversity of cellular responses, such as proliferation, differentiation, cell death, and survival [23, 24].

Of the three types of MAPKs, ERKs and JNKs have been recently shown to regulate the redirecting of energy harvest to glycolysis in both malignant and highly proliferative cells by affecting the activity of key metabolic regulators. Here we provide a comprehensive overview of the functional implications and our current knowledge of the role of ERK and JNK signaling pathways in regulating glucose metabolism of highly proliferating cells in cancer and some physiological contexts, such as inflammation and immunity as well as tissue development.

### The glycolytic pathway and its regulation

**Fig. 1:**
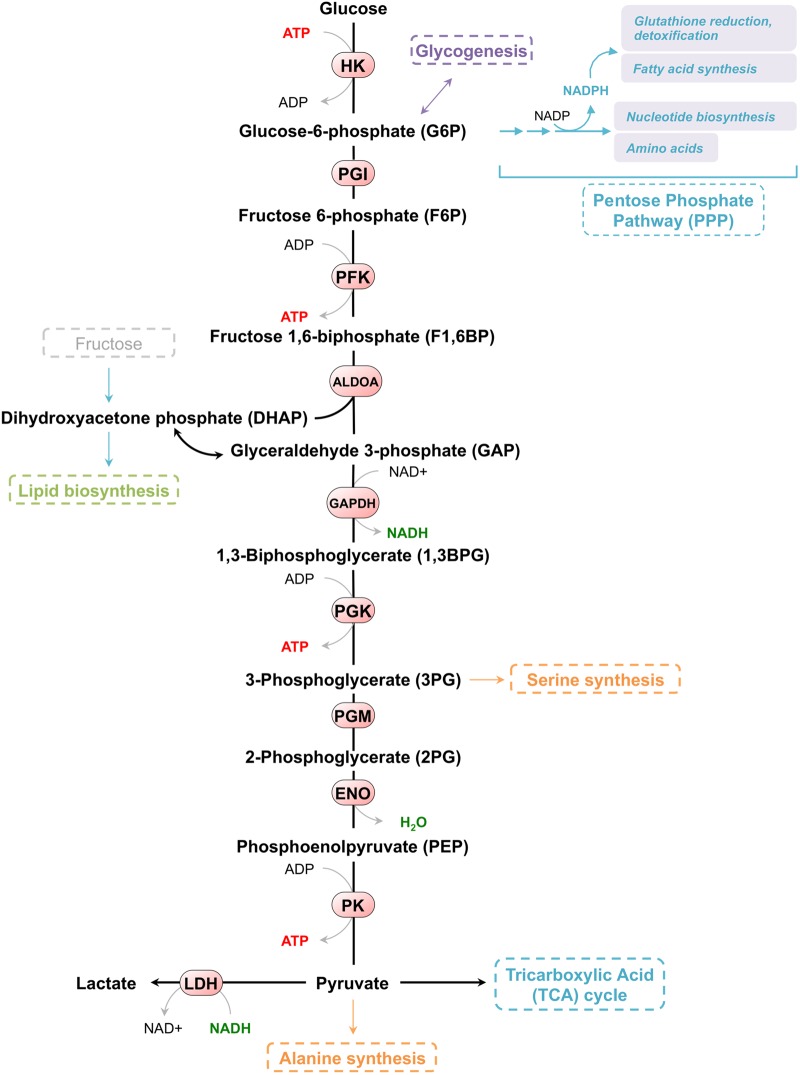
Schematic diagram of glycolysis. Schematic drawing shows the steps and specific enzymes of the glycolytic pathway that converts glucose in pyruvate through a series of enzymatic reactions catalyzed by hexokinase (HK), phosphoglucose isomerase (PGI), phosphofructokinase (PFK), aldolase (ALDOA), glyceraldehyde 3 phosphate dehydrogenase (GAPDH), phosphoglycerate kinase (PGK), phosphoglycerate mutase (PGM), enolase (ENO), and pyruvate kinase (PK). Lactate dehydrogenase (LDH) converts pyruvate in lactate. Shown are also the biosynthetic pathways that originate from glycolytic intermediates

Most mammalian cells use glucose as the primary carbon sources for the production of ATP and synthesis of cellular components, such as proteins, lipids, and nucleic acids [25–27]. They normally take up glucose from extracellular fluid into the cell only when stimulated by extracellular growth factors to growth and divide [28–31]. For example, the binding of growth factors to receptor tyrosine kinases (RTKs) activates the phosphatidylinositol 3-kinase (PI3K)/Akt pathway to stimulate cellular glucose uptake and glycolysis along with cell growth and survival [19, 32] by enhancing both the transcriptional expression and translocation to the cell surface of glucose transporters (GLUTs) [15, 16, 33]. Once in the cell, glucose is phosphorylated by the first enzyme of the glycolytic pathway hexokinase (HK) to form glucose-6-phosphate (G6P), which in turn serves as an allosteric inhibitor of HK (Fig. 1). HK is stimulated following activation of Akt, so that PI3K/Akt signaling not only facilitates the increase in glucose uptake but also enables glucose progression through the glycolytic pathway [17, 18, 31, 34]. G6P has then three possible metabolic fates within the cell. It can be converted into fructose-6-phosphate (F6P) in the glycolytic pathway or can be oxidized by the pentose phosphate pathway (PPP) or enter the synthesis pathway of glycogen, a storage form of glucose (reviewed in ref. [35]) (Fig. 1). If a cell is instructed to continue glycolysis, F6P is further phosphorylated by the enzyme phosphofructokinase 1 (PFK1) to form fructose 1,6-biphosphate (F1,6BP), which then is cleaved into two triose phosphates, glyceraldehyde 3-phosphate (GAP) and dihydroxyacetone phosphate (DHAP) (Fig. 1) [2, 3]. Unlike GAP, which is the substrate for the next reaction in glycolysis, DHAP does not undergo direct glycolysis. It can either be used to generate glycerol-3-phosphate (G3P), an important precursor for the synthesis of structural lipids of cell membranes, or can proceed further along the glycolytic pathway via its conversion to GAP by triose phosphate isomerase. As a result, oxidation of one molecule of glucose forms two molecules of GAP, both of which are converted into pyruvate in a sequence of five reactions that generates ATP and NADH (Fig. 1) [2, 3]. The final reaction in this sequence is the conversion of phosphoenolpyruvate (PEP) to pyruvate, which can then enter into TCA cycle by its conversion to acetyl-coenzyme A (acetyl-CoA) or be converted into lactate depending on cell type and availability of oxygen [2, 3]. The conversion of PEP to pyruvate is catalyzed by pyruvate kinase (PK), a homotetrameric enzyme that exists in mammals as four isoforms (PKL, PKR, PKM1, and PKM2) with different expression patterns and regulatory mechanisms [36–42]. Unlike PKM1, which is constitutively active and insensitive to allosteric effectors, the PKM2, PKL, and PKR isoforms are subject to allosteric regulation that affects enzyme activity by the direct binding of effectors. For example, the glycolytic intermediate F1,6BP stimulates PKM2 by increasing its affinity for PEP (the catalytic substrate of PK), promoting tetramerization, and stabilizing the tetrameric active conformation of PKM2. Conversely, PKM2 is inhibited by the binding to tyrosine phosphorylated peptides, which induce the release of F1,6BP resulting in the stabilization of the inactive dimeric conformation of PKM2, an event associated with low PK activity [36]. Besides being allosterically regulated by diverse metabolites, PKM2 is also negatively regulated by covalent modifications, including phosphorylation, acetylation, and oxidation. It is now appreciated that low PKM2 activity in cells allows the accumulation of glycolytic intermediates upstream of the PK reaction that can be used as precursors for the synthesis of nucleotides, amino acids, and fatty acids (Fig. 1) (reviewed in ref. [43]). Therefore, maintaining a low PKM2 activity is particularly important for highly proliferating cells, such as many cancer cells, that require a copious supply of nucleotides, amino acids, and lipids for biomass duplication. Paradoxically, the low level of PKM2 activity in rapidly proliferating cells is associated with an increased conversion of pyruvate into lactate with concomitant generation of NAD^+^ from NADH in an enzymatic reaction catalyzed by lactate dehydrogenase A (LDHA). As NAD^+^ is consumed by glyceraldehyde-3-phosphate dehydrogenase activity in a reaction that generates 1,3-biphosphoglycerate from GAP in glycolysis (Fig. 1), an efficient regeneration of NAD^+^ is required to maintain the continuity of the glycolytic flux [44]. Therefore, it appears that pyruvate is converted into lactate to sustain high glycolytic flux, regenerating NAD^+^. Interestingly, NAD^+^ is not only required to enable glycolysis but is also needed for nucleotide and amino acid biosynthesis pathways that branch from glycolysis [26, 44–47]. Therefore, during rapid cell proliferation, an efficient enzymatic activity of LDHA provides an advantage to cells by regenerating NAD^+^.

### The Warburg effect: a novel perspective

It has long been known that normal highly proliferating and tumor cells display the Warburg-like metabolic phenotype, which is characterized by high rate of glucose uptake and conversion to lactate under aerobic conditions [20, 44, 48]. One of the most important debates about this metabolic phenotype is that aerobic glycolysis is an inefficient metabolic pathway generating less ATP per single molecule of glucose than that generated through OXPHOS and, plainly, aerobic glycolysis cannot cope with the high cellular demand of energy required during fast cell proliferation. A number of possible explanations have been proposed to account for the Warburg metabolism of proliferating cells. One possible explanation is that aerobic glycolysis essentially generates more ATP by producing it at a faster rate than OXPHOS [25, 44]. Thus it appears that the theoretical inefficiency of energy generation of glycolysis is counterweighed by the rapid production of ATP. Additionally, an increase in glycolytic flux is believed to be advantageous for proliferating cells with high demand for reducing equivalents (such as NADPH) and cellular macromolecules (such as DNA, proteins, and lipids) [49]. This is because an accelerated glycolytic flux can lead to an accumulation of glycolytic intermediates, which can be channeled into biosynthetic pathways. For example, G6P, the first glycolytic intermediate, can be oxidized through the PPP to generate the nucleotide precursor ribose-5-phosphate and NADPH, which is used for lipid biosynthesis and scavenging of reactive oxygen species (ROS) produced during fast cell proliferation (Fig. 1) [26, 32, 50]. Similarly, DHAP and 3PG, other two glycolytic intermediates, can leave the glycolytic flux and participate in the phospholipids and serine biosynthesis pathway, respectively (Fig. 1). It is important to note, however, that these glycolytic intermediates will not accumulate and branch off into their respective biosynthetic pathways unless the final enzymatic reaction in glycolysis (the conversion of PEP into pyruvate) is slowed down. To achieve this, proliferating cells and many cancer cell types predominantly express and use PKM2, which, as discussed above, has a low PK activity and therefore is less efficient in converting PEP to pyruvate than PKM1, thereby allowing for upstream glycolytic intermediates to accumulate and branch off into biosynthetic pathways. As such, PKM2 expression and its low activity is known to promote cancer cell proliferation [36–42], although recent studies in mouse cancer models have led to opposing conclusions [51]. For example, absence of PKM2 accelerates tumor formation in a *Brca1*-loss-driven model of breast cancer [40], in a mouse model of medulloblastoma [52], and results in spontaneous hepatocellular carcinoma (HCC) development in aged mice [53], indicating that PKM2 negatively regulates tumorigenesis. However, other in vivo studies support the notion that PKM2 has as an oncogenic function in leukemia [54] and soft tissue sarcoma formation [55]. It therefore seems likely that the function of PKM2 in cancer development depends on the cancer type. Taken together, the findings described above support the hypothesis that increased aerobic glycolysis is a metabolic strategy to improve the availability of NADPH and metabolic substrates needed for rapid biomass synthesis during fast cell proliferation. Increases in the rates of glycolysis have also beneficial antioxidant role for cells, generating NADPH essential for protecting cells from oxidative damage driven by increased cell proliferation (Fig. 1) [44, 56].

**Fig. 2:**
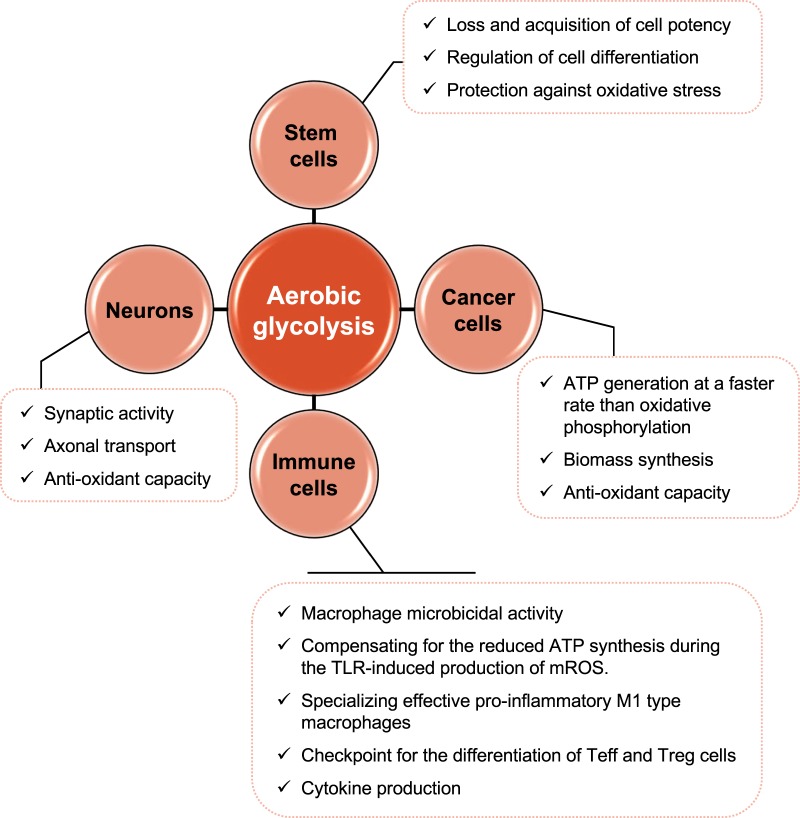
Cellular functions associated with aerobic glycolysis: to proliferation and beyond. Aerobic glycolysis has been widely linked to cell proliferation, especially in cancer cells where it serves to generate sufficient energy (by means of ATP) and synthesis of building blocks needed for cell growth and division. Aerobic glycolysis provides also antioxidant capacity to many different cells (i.e., cancer cells, immune cells, neurons, and stem cells) to protect against oxidative stress-induced apoptosis and provide survival advantages. Other than serving as an antiapoptotic pathway, aerobic glycolysis is crucially required for specific cellular functions: (i) biosynthesis of neurotransmitters, (ii) activation and differentiation of specialized cells, (iii) antimicrobial activity, and (iv) naive to primed pluripotency

Although the Warburg effect has now been widely accepted as an emerging hallmark of cancer, it is a distinctive feature of many highly proliferating normal cells and fulfills a number of homeostatic functions, which include brain functionality, immune responses, and tissue remodeling in embryogenesis (Fig. 2) [56–62]. Thus the Warburg-like glucose metabolism has probably evolved not only to satisfy the specific biosynthetic needs of any differentiated cell type during rapid proliferation but also to regulate cell fate and functions (Fig. 2). Because of their increased biosynthetic needs, many types of cancer cells, unlike their normal counterparts, adopt this tightly controlled metabolic strategy to support their own deregulated proliferation. Therefore, targeting enhanced glycolysis in cancer represents a worthwhile therapeutic strategy. In this respect, it worth to note that direct inhibition of metabolic enzymes could cause adverse cellular effects and unwanted toxicity because of their relevance to normal physiological functions, such as immunity and brain development [56–62]. Therefore, effective treatment of highly glycolytic tumors will require maintaining a delicate balance between suppressing deleterious functions of glycolytic enzymes and interfering with cellular physiology (Fig. 2). Treatments aimed at inhibiting specific isoforms of certain glycolytic enzymes, the expression of which is associated with cancer, or targeting metabolic enzymes in a deregulated metabolic pathway specific to cancer cells may have better therapeutic efficacy and reduce undesired side effects. We refer the interested readers to dedicated reviews for a detailed discussion of few examples of such metabolic inhibitors that have shown promising outcomes in animal models [35, 63–65]. Therefore, a better understanding of how the Warburg-like metabolism is regulated in normal physiological contexts could lead to more effective ways of targeting metabolic pathways without toxicity (Fig. 2).

### The Warburg effect in normal cellular functions

#### The role of the Warburg effect in stem cells

Research on stem cell biology recently provided compelling evidence in support of a role for aerobic glycolysis in the regulation of cell differentiation in various cellular contexts (Fig. 2) [66–72]. During the cellular differentiation program, pluripotent embryonic stem cells (ESCs) proceed through several stages, becoming more specialized (differentiated) at each step. Recent investigations indicate that ESCs display elevated rates of glycolysis with lactate production before differentiation and gradually shift toward OXPHOS as they mature and become terminally differentiated [66–69]. For example, Harris and colleagues [69], using embryonic *Xenopus* retinal tissue, demonstrated that dividing retinal progenitors are more reliant on aerobic glycolysis when compared with more differentiated cells. A similar switch from glycolytic to oxidative metabolism accompanied by increases in the expression of mitochondrial genes has been reported to be essential for the differentiation of murine ESCs to cardiomyocytes [70, 71], as well as human neuronal progenitor cells to differentiated neurons [66]. Although the exact mechanisms underlying such metabolic shift during cell differentiation is not fully clear, recent works have proposed several potential mechanisms. In the embryonic heart, immature cardiomyocytes display an open mitochondrial permeability transition pore (mPTP) and a glycolytic phenotype [72]. Pharmacologic and genetic closing of the mPTP cause structure and function maturation of mitochondria and result in accelerated cardiomyocyte differentiation, suggesting that mPTP dynamics regulate cardiomyocyte differentiation. Moreover, gene-targeting studies in mice revealed a key role for the nuclear receptor peroxisome proliferator-activated receptor-α (PPAR-α) and its cardiac-enriched coactivator protein, proliferator-activated receptor γ-coactivator (PGC)-1β, inducing the expression of mitochondrial genes and suppressing the expression of GLUT and glycolytic enzymes during cardiomyocyte maturation from fetal to adult [73].

In contrast, a metabolic switch from oxidative to glycolytic metabolism with high levels of lactate production has been found to take place during reprogramming of somatic cells to induced pluripotent stem cells (iPSCs), in vitro [74, 75]. Differentiated somatic cells are highly dependent on mitochondrial OXPHOS for energy production and need a switch to glycolysis when they enter a pluripotent state through reprogramming. Recent studies demonstrated that such switch, which is accompanied by an upregulation of glycolytic genes, precedes the expression of pluripotent genes during the reprogramming [74, 75]. This suggests that a Warburg-like metabolic phenotype is important for the acquisition of pluripotency, the ability of a stem cell to differentiate into any cell type of the adult body. In line with this, inhibition of glycolysis via various pharmacologic means attenuates the somatic cell reprogramming to iPSC, whereas the induction of aerobic glycolysis enhances the efficiency of iPSC generation [74–77]. Thus it appears that an increase in glycolysis accompanied by a low mitochondrial activity drives the somatic cell reprogramming process. Mechanistically, two transcription factors, hypoxia-inducible factors (HIFs) and c-Myc, the main positive regulators of aerobic glycolysis in cancer [13, 21, 30], have emerged as factors essential for the maintenance and acquisition of a pluripotent state [76, 77]. Taken together, the findings described above indicate that shifts between glycolysis and mitochondrial OXPHOS are intertwined with cell differentiation and reprogramming to pluripotency.

#### The role of the Warburg effect in the immune response

Similar to cancer cells, inflammatory immune cells such as M1 type macrophages, neutrophils, and dendritic cells exhibit a metabolic shift to glycolysis when activated through Toll-like receptors (TLRs) upon pathogen recognition [56–61, 78]. This leads to the production of various pro-inflammatory cytokines including interleukin-1β (IL-1β) and tumor necrosis factor-α (TNF-α). This metabolic shift involves an increase in the expression of GLUT1 (*SLC2A1*) gene and specific glycolytic genes as well as elevated lactate production accompanied by a decline in mitochondrial activity. Additionally, flux into the PPP, which allows the synthesis of nucleotides and NADPH generation, also enhances [57, 58, 79, 80]. It is now appreciated that the glycolytic metabolism allows mature activated immune cells to sustain rapid ATP production and satisfy the high demand of biosynthetic precursors associated with an acute inflammation or antibacterial response. Indeed, unlike cancer cells, activated immune cells are not highly proliferative, implying that a high proliferation rate is not the only explanation for why aerobic glycolysis is selected for in immune cells upon TLR activation [78]. In TLR-activated macrophages, for example, this metabolic choice may reflect the important role of mitochondrial ROS (mROS) in their bactericidal activity [58, 81, 82]. mROS are generated when electrons prematurely exit the electron transport chain and incompletely reduces oxygen to form superoxide (O_2_^–^), thus compromising the mitochondrial synthesis of ATP. It seems, therefore, that one potential benefit of favoring glycolysis over mitochondrial OXPHOS for ATP production would be to compensate for reduced mitochondria ATP production as mitochondria is used to produce ROS for the clearance of intracellular bacteria [58, 81, 82]. More recently, the metabolic shift toward the glycolytic pathway has been shown to be essential for the migration of activated macrophages to the sites of inflammation. Pharmacological inhibition of the glycolytic pathway or uptake of glucose suppresses the migration of murine macrophages to inflamed tissue. Moreover, PKM2 was found to localize in filopodia and lamellipodia, two cytoskeleton structures essential for cell migration [83]. This indicates that glycolysis is positively associated with the migratory properties of macrophages.

The important role for the glycolytic pathway in immune responses was also revealed by studies on T cells that carry the CD4 antigen. In the presence of specific cytokine microenvironment, this specialized population of T cells (CD4+ T cells) become activated and can differentiate into either effector T cells (Teffs) or regulatory T cells (Tregs) following the engagement of the T-cell antigen receptor (TCR) and co-stimulatory receptors [59, 60]. Whereas Teffs, which include T helper type 1 (Th1), Th2, and Th17 cell subset, are involved in the inflammatory responses, induced Tregs (iTregs) limit inflammation and possess immunoregulatory functions [59, 60]. It is now clear that Teffs and iTregs adopt distinct metabolic programs to attain their opposing functions, with Teffs expressing high surface levels of the GLUT1 and being highly glycolytic, whereas Tregs express low levels of GLUT1 and exhibit oxidative metabolism [84]. Importantly, inhibition of glycolysis has been shown to block the development of Th17 cell subset and at the same time to promote Tregs generation, indicating that glycolysis is crucial for controlling T cell lineage choices [58, 85, 86]. Moreover, glycolysis is not required to promote proliferation and survival of T cells but is needed instead for T cell migration and effector functions as well as antitumor immunity [58, 60, 87].

The close relationship between glycolysis and antitumor immunity has received considerable attention in the past few years since the reported success of adoptive T cell immunotherapy [88]. Insufficiency of glucose in the tumor microenvironment caused by high rates of glucose uptake in tumor cells has been shown to suppress the antitumor response of tumor-infiltrating T cells, indicating a mechanism by which glycolytic metabolism of tumor cells directly suppresses the antitumor T cell function. More importantly, enforcing the production of the glycolytic metabolite PEP in such suppressed infiltrating T cells restored the effectiveness of their antitumor responses, which resulted in suppression of tumor growth upon adoptive transfer [89]. Thus glycolysis seems to have an important contribute in the choice between pro-inflammatory and anti-inflammatory CD4+ T cell subsets and their antitumoral functions. As such, manipulating the glycolytic pathway in tumor and/or T cells may be beneficial in enhancing the efficacy of adoptive cancer immunotherapy. Along these lines, the swift to glycolysis has also been shown to be a critical event in M1 and M2 macrophage polarization, a tightly controlled process by which macrophages acquire distinct phenotypes and functional capabilities in response to diverse tissue-derived micro-environmental signals [90]. While M1 macrophages have an inflammatory phenotype with a strong antimicrobial and antitumor activity, M2 macrophages prevent excess inflammation and promote tissue repair and remodeling as well as antiparasitic immunity and tumor progression [91]. It is worth noting that a shift in the balance between these polarization states of macrophages is central to a spectrum of human diseases, including obesity and cancer. For example, diet-induced obesity has been shown to be a consequence of an inappropriate accumulation of proinflammatory M1 state in the adipose tissue that leads to insulin resistance [92], whereas chronic weight loss was found to result from an excess presence of M2 macrophages in the adipose tissue [93]. Moreover, a high density of tumor-associated macrophages, which closely resemble the M2 state, has been shown to associate with tumor progression and poor prognosis in various tumour types [94]. Interestingly, inflammatory M1 macrophages favor glycolysis over mitochondrial OXPHOS for rapid pathogen killing, whereas anti-inflammatory M2 macrophages use OXPHOS as the main ATP-producing pathways [58, 60]. As such, blocking mitochondrial ATP production with oligomycin resulted in phenotypic repolarization of M2 to M1 cells [95]. Therefore, broader understanding of the metabolic features of M1 and M2 macrophages could indicate new targets for manipulating macrophage polarization in a therapeutic context. Taken together, the findings described above illustrate the importance of the Warburg effect in promoting the effector functions of immune cells (Fig. 2). Importantly, the signaling mechanisms that regulate aerobic glycolysis are the subject of intense ongoing research. Several studies showed that activation of certain signaling pathways, such as ERK and JNK pathways, can directly or indirectly affect the transcriptional or posttranscriptional regulation of enzymes involved in glycolysis and OXPHOS as well as their anabolic pathway branches. A stepwise description of the roles of these two MAPK pathways in promoting the Warburg effect is outlined in the following sections.

### The ERK signaling and the Warburg effect

**Fig. 3:**
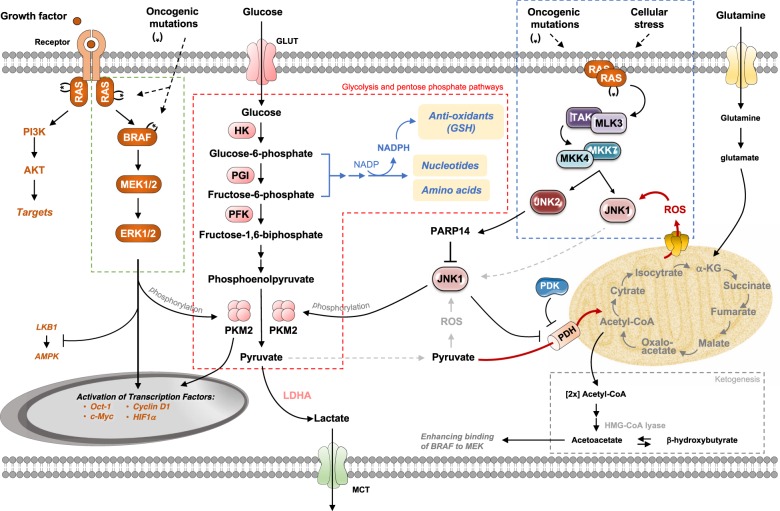
The control of aerobic glycolysis by ERK and JNK signaling pathways in proliferating cells. Glycolysis (red-dotted shape) starts when glucose enters the cells through GLUTs and is converted into glucose-6-phosphate by the first glycolytic enzyme hexokinase (HK). The final product of glycolysis is pyruvate. Its production is tightly regulated by the glycolytic enzyme PKM2, whose activation, conformational state, and cellular localization is tightly regulated by posttranslational modifications, which includes phosphorylation, cysteine oxidation, and acetylation. Pyruvate could be further oxidized in the mitochondrion through its conversion to acetyl-CoA for subsequent oxidation in the tricarboxylic acid (TCA) cycle. The shunting of pyruvate into the mitochondrion is regulated by the activity of pyruvate dehydrogenase (PDH), which in turn is negatively regulated by pyruvate dehydrogenase kinases (PDKs) under hypoxia. In proliferating cells, a largest amount of pyruvate is converted to lactate contributing to the Warburg effect. The formation of lactate, catalyzed by lactate dehydrogenase (LDH), is necessary for the rapid regeneration of NAD^+^ from NADH, which is then reused to maintain active the glycolytic flux. Aerobic glycolysis of cells in multicellular organisms is regulated by both extracellular and intracellular signaling pathways. Engagement of growth factors to their receptors signals activation of PI3K/AKT pathway and the phosphorylation cascade of RAS/BRAF/MEK/ERK (green-dotted shape). The BRAF/MEK/ERK signaling cascade can be also activated by oncogenic mutations and culminates with activation and translocation of ERK to the nucleus, which regulates the expression and activity of transcription factors that directly control the expression of glycolytic enzymes in cancer cells. This network of transcription factors, including hypoxia-inducible factor-1α (HIF-1α) and c-Myc, drives the Warburg effect downstream of oncogenic BRAF(V600E) mutation in melanomas. Binding and activation of MEK by BRAF is further enhanced after accumulation in the cytoplasm of acetoacetate, a byproduct of ketogenesis—a biochemical process by which cells produce ketone bodies by the breakdown of fatty acids and ketogenic amino acids such as glutamine (gray-dotted shape). RAS-mediated oncogenesis and cellular stress also contribute to the activation of JNK cascade (blue-dotted shape). Once activated, upstream MAP3K kinases (e.g., TAK1 and MLK3) phosphorylate and activate MKK4 and MKK7, which in turn phosphorylate and stimulate the activity of distinct JNK isoforms. Upon activation, each JNK protein delivers different cellular activities. While JNK1 negatively regulates aerobic glycolysis via direct phosphorylation of PKM2 and PDH, JNK2 positively controls aerobic glycolysis via upregulation of PARP14, a direct inhibitor of JNK1-mediated phosphorylation of PKM2 in cancer cells. Notably, JNK1 activation depends also on the formation and accumulation of mitochondrial and cellular ROS

In addition to their recognized role in controlling cell proliferation and survival, many of the signaling pathways downstream of both oncogenes and tumor-suppressor genes can regulate the glucose metabolism [6, 32]. For example, the ERK-MAPK signaling pathway, which is activated by the RAS oncoproteins (HRAS, KRAS, and NRAS) and positively associated with cell proliferation and survival [23, 24], has been shown to promote the Warburg effect [96]. Like other MAPK signaling pathways, the ERK pathway is activated by a series of phosphorylation events (i.e., the MAPK model) that occur downstream of a variety of activated receptor types including RTKs in response to extracellular stimuli such as growth factors (Fig. 3). The initiating kinases (i.e., the MAP3K) are members of the RAF family, which include ARAF, BRAF, and CRAF, and often activated as a result of their interaction with active GTP-bound RAS proteins. This interaction, which occurs at the inner leaflet of the plasma membrane, leads to the formation of active homodimers or heterodimers of the RAF protein kinases (Fig. 3). Once activated, RAF kinases phosphorylate and activate components of the MAP2K module, such as MEK1 and MEK2, which in turn, activate the two MAPK protein kinases, ERK1 and ERK2, through phosphorylation of both tyrosine and threonine residues present in a conserved tripeptide motif (*Thr-Glu-Tyr*) within their activation loop. Upon activation, ERK1 and ERK2 phosphorylate and activate a large number of nuclear and non-nuclear proteins, including transcription factors of the ETS family, the ternary complex factor transcription factors, c-Myc, signal transducer and activator of transcription factor 3, nuclear factor (NF) of activated T cells, as well as cell survival regulators of the BCL-2 protein family in the mitochondria [23, 24]. These proteins regulate a diversity of cellular processes, such as cell proliferation, growth, survival, differentiation, and motility, whose deregulation has been associated with cancer [23, 24, 97].

Indeed, constitutive activation of ERK1 and ERK2 signaling is frequently observed in human cancers due to mutations in genes that encode RTKs, RAS, BRAF, CRAF, MEK1, and MEK2 [24, 98]. In melanoma, for example, up to 70% of these tumors have point mutations in the BRAF gene, the majority of which lead to a single amino acid substitution of valine for glutamic acid at position 600 (the BRAFV600E mutation). These mutations favor the active structural conformation of BRAF kinase, causing the constitutive activation of ERK1/2 pathway, which then activates proliferative programs and promotes the aerobic glycolytic phenotype via induction of transcriptional regulators of glycolysis, the TCA cycle, and macromolecular biosynthesis (Fig. 3) [98]. Among these transcription factors are c-Myc, which increases the expression of GLUT1, LDHA, and a number of enzymes in the glycolytic pathway, as well as HIF-1α, which also upregulates LDHA and cooperates with c-Myc in the induction of HK2 [99–101]. c-Myc is also known to induce expression of enzymes involved in nucleotide and fatty acid synthesis as well as glutaminolysis, which sustains the pool of metabolic intermediates in the TCA cycle that in turn can be used as biosynthetic precursors to generate amino acids and fatty acids for anabolic growth [102–104]. Moreover, c-Myc facilitates the glycolytic intermediates flux to the PPP, serine, and glycine biosynthesis pathways by promoting PKM2 expression [105]. In line with this, enforced expression of BRAF(V600E) in melanoma cells has been shown to upregulate the expression of glycolytic and PPP enzymes to sustain melanoma cell growth and proliferation [106]. Interestingly, Haq and collaborators [107] observed that BRAF(V600E) expression in melanomas correlates with decreased expression of oxidative enzymes, diminished mitochondrial number and function, and increased production of lactate. The authors also showed that activated BRAF/ERK pathway promotes glycolytic phenotype in melanoma cells by downregulating the expression of the mitochondrial biogenesis and function factor, PGC-1α, thereby inhibiting the mitochondrial oxidation [107].

In addition, high levels of serum lactate were observed in patients with BRAF mutant melanomas [108], providing evidence of linking oncogenic BRAF/ERK signaling to aerobic glycolysis in a clinical setting. Activating mutations in the BRAF gene have also been identified in non-melanoma tumors, including thyroid, colorectal carcinomas, lung cancer, and hairy cell leukemia [109], and were linked to the glycolytic phenotype in both in vitro and in vivo cancer models [110, 111].

The link between the Warburg effect and the RAF/MEK/ERK pathway was further confirmed by cellular and xenograft studies using BRAF inhibitors or MEK inhibitors. A decrease in the expression levels of various glycolytic genes, including GLUT1, GLUT3, and HK2, lactate and ATP production was observed in a panel of BRAF(V600E) melanoma cell lines treated with the BRAF inhibitor vemurafenib as well as in samples from patients undergoing BRAF inhibitor therapy. Such effect was associated with a decrease in the transcription of ERK1/2 target genes [97]. Importantly, treatment of vemurafenib-resistant BRAFV600E melanoma cells with vemurafenib in combination with the pyruvate mimetic dichloroacetate, which inhibits glycolysis as a consequence of an increase in glycolysis-derived pyruvate flux into the TCA cycle [112], was shown to restore the expression of glycolytic enzymes and re-sensitize these resistant cells to vemurafenib [98], indicating that glycolysis contributes to resistance to BRAF/MEK/ERK pathway inhibition in melanoma. A decrease in glucose uptake, lactate levels, and HK2 expression was also observed in human cancer cells harboring mutant BRAF and BRAF-driven melanoma xenografts following MEK1/2 inhibition [113], confirming a positive correlation between the glycolytic phenotype of cancer cells and BRAF/MEK/ERK pathway activation.

An important role of ERK1/2 signaling in promoting the Warburg-like metabolism can also be inferred from studies in other oncogenic contexts. DePinho and colleagues [114], using an inducible mouse model of pancreatic cancer driven by the *Kras* oncogene, demonstrated that oncogenic activation of the RAF/MEK/ERK pathway sustains tumor growth by inducing transcriptional upregulation of key genes that promote both the uptake and consumption of glucose to produce lactate, resulting in an increase in glycolytic intermediates flux into anabolic pathways, such as the hexosamine and non-oxidative PPP pathways, which provide precursors for protein glycosylation and nucleotide biosynthesis. The ERK-directed transcriptional program was found to be dependent on c-Myc transcriptional activity, providing further evidence linking the ERK1/2 signaling to the Warburg-like metabolism in cancer cells [114]. Another intriguing link between the ERK1/2 signaling and cancer-associated Warburg effect is provided by the glycolytic enzyme phosphoglycerate kinase 1 (PGK1) [115]. Activation of ERK1/2 by hypoxia, epidermal growth factor (EGF) stimulation, mutant BRAF, or KRAS was shown to induce mitochondrial translocation of PGK1, through phosphorylation of S203. This phosphorylation event in turn results in phosphorylation and activation of pyruvate dehydrogenase kinase (PDK), of which there are four isoforms (PDK1–4) [115]. Upon activation, PDK1 inhibits the enzyme complex pyruvate dehydrogenase (PDH), which converts pyruvate into acetyl-CoA (the main substrate for the TCA cycle), resulting in the suppression of pyruvate consumption and ROS production in mitochondria and increased lactate production. Thus, by promoting PGK1 mitochondrial translocation, ERK1/2 enhances aerobic glycolysis and compromises the TCA cycle, resulting in brain tumorigenesis (Fig. 3) [115]. In another study, however, oncogenic activation of ERK1/2 was shown to positively regulate TCA cycle flux (via PDH) by suppressing the PDK4 expression. The positive regulation of PDH flux by ERK1/2 signaling was associated with an increase in cell proliferation rates [116]. Given that the TCA cycle supplies substrates for energy production by OXPHOS and intermediates for lipid and amino acid synthesis [32, 103], these contrasting findings probably reflect the fact that proliferating cells modulate PDH flux through ERK1/2 signaling to suit distinct metabolic needs of each specific cell type.

Besides affecting metabolic enzymes, ERK1/2 pathway can also influence cellular metabolism indirectly by controlling the AMP-activated protein kinase (AMPK), a key regulator of energy homeostasis that is activated under low-energy conditions by the tumor-suppressor liver kinase B1 (LKB-1) in most cellular contexts [117]. Upon activation, AMPK inhibits almost all biosynthetic pathways needed for cell proliferation to decrease ATP consumption and activates ATP-producing catabolic pathways, thus allowing cells to restore energy homeostasis [117]. Although activated AMPK has been shown to enhance glucose uptake and glycolysis in certain contexts [118], mouse embryo fibroblasts or cancer cells lacking AMPK activity exhibit an elevated glucose consumption and lactate production associated with increased lipid biosynthesis and ability to form tumors in vivo [119, 120]. Mechanistically, the transcription factor HIF-1α has been shown to be required for increased glycolysis and biosynthesis observed in AMPK-deficient cells. In line with recent studies in human gastric cancer cell [121], these observations suggest that AMPK can suppress the Warburg-like metabolism that underpins tumorigenesis. In support of this view, downregulation of LKB1-AMPK signaling by oncogenic signaling pathways that promote the Warburg-like phenotype has been reported in many cancers [119]. In BRAF(V600E) melanoma cells, for example, activated ERK has been shown to phosphorylate LKB1, rendering this enzyme unable to bind to and activate AMPK [122, 123] (Fig. 3). The inactivation of LKB1 by ERK has been shown to be instrumental in BRAF(V600E)-driven tumorigenesis. The inhibition of the LKB1-AMPK axis by ERK was also shown to promote cell growth and proliferation in other highly glycolytic cancers [123], providing further evidence linking ERK1/2 signaling to the metabolic features of cancer cells.

**Fig. 4:**
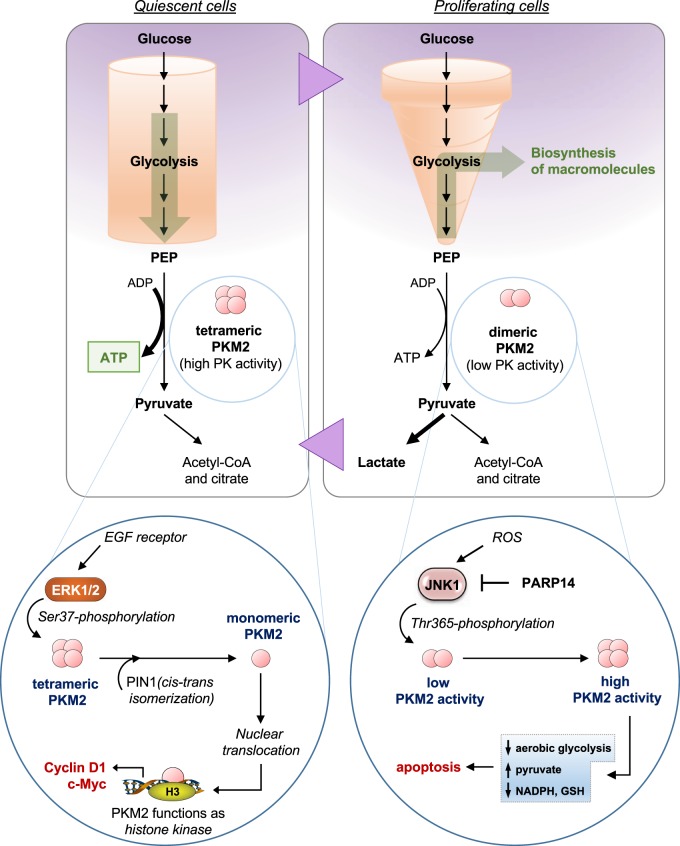
ERK- and JNK-mediated phosphorylation of PKM2 is at the crossroad between proliferation and apoptosis. PKM2 acts as master regulator of the Warburg effect. Of the many posttranslational modifications, phosphorylation of PKM2 by either ERK or JNK1 dictates distinct outcomes. In quiescent cells, PKM2 is present as a tetrameric protein associated with elevated enzymatic activity. When cells receive a growth stimulus, activation of ERK drives phosphorylation of tetrameric PKM2. Phosphorylated PKM2 is then *cis-trans* isomerized by PIN1 allowing dissociation of tetrameric PKM2 to monomers. Monomeric PKM2 enters the nucleus where it acts as histone-binding protein allowing gene expression regulation of both glycolytic enzymes and cell cycle regulators (i.e., c-Myc, cyclin D1). Besides, accumulation of reactive oxygen species (ROS) in the cytoplasm promotes activation of JNK1, which can phosphorylate and enhance PKM2 activation, allowing cells to reduce their antioxidant capacity and induce apoptosis. Notably, enhanced expression of PARP14 in cancer cells suppresses JNK1-mediated phosphorylation and activation of PKM2, providing therefore survival advantages to cancer cells. PARP14 by suppressing JNK1 activity contributes to maintain low PKM2 activity and, combined with a robust glycolysis, leads to an accumulation of glycolytic intermediates, including precursors of nucleic acids, lipids, and amino acids. This accumulation provides a metabolic bottleneck allowing glycolytic intermediates to be redirected toward biosynthesis, fueling through the pentose phosphate pathway for DNA synthesis and thereby contributing to the rapid cell proliferation seen in tumors

Constitutive activation of ERK1 and ERK2 by mutational activated RTKs, such as the EGF receptor mutant III (EGFRvIII), also leads to the Warburg effect phenotype. Lu and colleagues [96] demonstrated that EGFR-activated ERK1/2 binds to and phosphorylates PKM2 at Ser37 favoring its nuclear translocation (Fig. 4). Importantly, ERK1/2 phosphorylates PKM2, but not PKM1, leading to Pin1-dependent *cis-trans* isomerization and conversion of PKM2 from a tetramer to a monomer. In the nucleus, PKM2 couples with transcriptional factors and functions as a protein kinase that phosphorylates histone H3 for gene transcription of cyclin D1 and c-Myc, which promotes the expression of glycolytic enzyme genes (Fig. 4) [96].

However, it is worth noting that c-Myc not only promotes glycolysis but also favors mitochondrial respiration by enhancing the expression of genes involved in mitochondrial structure and biogenesis [104, 124–126]. Indeed, knockdown of c-Myc in breast cancer cells with stem-like features was found to be associated with decreases in mitochondrial mass and oxygen consumption as well as in the number of mitochondria. The impaired mitochondrial function was associated with reduced mammosphere formation, which is an assay method to test “stemness” of cancer cells in vitro and tumor initiation in vivo [127, 128]. Thus both glycolysis and OXPHOS appear to be positively regulated by c-Myc and essential for tumorigenesis. As a whole, these examples illustrate that oncogenic alterations in ERK1/2 signaling pathway, which has a crucial role in sustaining proliferative programs, determine a metabolic switch from mitochondrial metabolism to glycolysis in cancer cells, fulfilling the energetic and biosynthetic requirements for tumor growth. As such, several therapeutic approaches targeting this pathway at multiple levels are currently being tested in clinical trials or used in the clinic for cancer treatment [129], and their efficacy have been correlated with the inhibition of glycolysis and/or anabolic metabolism [130–132].

There is also striking evidence that ERK1/2 activation is critical for the switch from OXPHOS to glycolysis observed in activated T cells that, as discussed above, is essential for T cell effector differentiation and function. Indeed, pharmacologic inhibition of ERK1/2 activity blocked the increase in glucose uptake and glycolysis as well as mRNA expression and activity of the glycolytic enzyme HK induced by the ligation of the TCR and the co-stimulatory receptor CD28 [133]. Furthermore, inhibition of ERK1/2 has been shown to impair glucose consumption and lactate production in macrophages activated by LPS. Mechanistically, the decrease in glycolysis appears to be related to the reduction in the levels of the glycolytic intermediate fructose-2,6-bisphosphate (F2,6BP) [134], an allosteric activator of the glycolytic enzyme PFK1 [6]. Thus, by enhancing glycolysis, ERK1/2 signaling positively controls T cell and pro-inflammatory macrophage function.

### The JNK signaling and the Warburg effect

Another MAPK subfamily mechanistically linked to the Warburg effect is the JNK kinase family [102], which includes three proteins (JNK1, JNK2, and JNK3) that are encoded by three separate genes, namely, *jnk1 (mapk8)*, *jnk2 (mapk9)*, and *jnk3 (mapk10)* [24]. Whereas JNK1 and JNK2 are ubiquitously expressed in mammalian cells, the expression of JNK3 is restricted to certain tissues [24]. The JNK proteins—also known as stressed-activated protein kinases—are activated by a variety of extracellular stimuli, including stress, proinflammatory cytokines, growth factors, pathogens, toxins, and drugs. Similarly to ERK1/ERK2, activated JNKs can directly phosphorylate a variety of cytoplasmic and nuclear substrates, which participate in a diversity of cellular processes, including proliferation, differentiation, apoptosis, and survival [135].

It is now appreciated that the functions of each JNK proteins can either differ or overlap depending on the cell type [135]. With respect to cancer, for example, JNK2 appears to be a crucial tumor promoter of carcinogen-induced skin cancer in contrast to JNK1 [136]. Moreover, JNK2, but not JNK1, is required for the survival of myeloma cells [137] and promotes the tumorigenicity of glioblastoma cells [138]. Conversely, JNK1 is required for proliferation of hepatocytes and liver cancer cells in vivo, while JNK2 appears to be dispensable [139]. Thus JNK proteins play different and even opposing roles in cancer development, although functional redundancy between JNK1 and JNK2 has also been reported [135]. Support evidence for the latter stems from studies showing that loss of either JNK1 or JNK2 has no effect on development of B lymphomas induced by transgenic expression of the c-Myc oncogene and overall survival rate of c-Myc-transgenic mice, thus indicating that loss of one JNK protein is compensated for by the other remaining [140]. Redundant or partially redundant roles for JNK1 and JNK2 proteins have also been identified in studies of Burkitt’s lymphoma [137, 141] and breast cancer cell lines, as well as mouse model of breast cancer caused by loss of a single allele of the *p53* tumor-suppressor gene [142, 143]. While the findings outlined above indicate that JNK proteins can play roles in tumor development, they also emphasize that JNK1 and JNK2 have either distinct or redundant functions. Whether JNK1 and JNK2 differentially affect cancer-associated metabolic changes is a matter of active investigation.

Despite a connection between JNKs and metabolic disorders (i.e., obesity-induced immune cell recruitment, inflammation in adipose tissue, insulin resistance, impaired glucose homeostasis) in mammalians have been widely described (reviewed in ref. [144]), very little is known about possible links between the JNK pathway and the metabolic reprogramming of tumor cells. Recent work from our group as well as several other laboratories revealed a role for the JNK pathway in restraining aerobic glycolysis to promote apoptosis in cancer cells [145]. In HCC cells, for example, JNK1 activity is suppressed by the antiapoptotic protein poly(ADP-ribose) polymerase 14 (PARP14), and this suppression appears to be the key determinant for the Warburg-like phenotype needed for enhanced HCC cell survival [146]. The inhibition of JNK1 by PARP14 was also shown to support antioxidant capacity of HCC cells by increasing NADPH and glutathione levels. At a mechanistic level, JNK1 stimulates PKM2 activity by enhancing the affinity of PKM2 for its substrates, PEP and ADP, through phosphorylation of Thr365.

This function of JNK1 seems to be one of possible mechanisms underlying the anti-Warburg effect and apoptotic role of JNK1 in cancer (Fig. 4) [146].

Moreover, it was shown that activation of the JNK pathway by the histone methyltransferase inhibitor chaetocin, which induces apoptosis in cancer cells by inducing ROS production, resulted in a reduction of glucose uptake and lactate production in glioma cells [147]. Although the mechanisms have not been explored in depth, JNK activation in glioma cells following chaetocin treatment led to a marked increase in PK activity and decrease of HK2 activity and expression [147], implying a role for the JNK pathway in restraining the glycolytic metabolism in glioma cells. Importantly, culturing cancer cells with elevated concentrations of pyruvate increased the activity of JNK1, but not JNK2, by enhancing ROS production [148]. Mechanistically, it was shown that activation of the ROS→JNK1 axis activates the ribosomal kinase p70S6K, which in turn suppresses glycogen synthase kinase-3β resulting therefore in augmented activity of glycogen synthase, an enzyme involved in converting glucose to glycogen, and subsequent diverting glucose away from the mitochondria [148]. Likewise, glutamine deprivation in osteosarcoma cells stimulates endoplasmic reticulum stress, which leads to the activation of JNK driving transcription and secretion of IL-8, needed for osteolysis associated with metastatic breast cancer [149]. Altogether these observations suggest the JNK pathway is involved in the regulation of cellular metabolism in cancer cells. In addition to cancer cells, JNK1 has been shown to suppress glycolysis in normal tissue. Knockdown of JNK1 in normal liver cells upregulated the hepatic expression of clusters of genes involved in glycolysis and in triglyceride synthesis pathways, suggesting that basal activity of JNK1 negatively regulates hepatic glycolysis and biomass formation [150]. This was further confirmed by studies using high-fat-fed mice with compound deficiency of JNK1 and JNK2 in hepatocytes, which exhibited increased expression of glycolytic enzymes and lactate production accompanied by a reduced rate of mitochondrial oxygen consumption. Such effects of JNK1 and JNK2 deletion were associated with an upregulation of genes involved in the PPAR, leading to marked increases in the rate of fatty acid oxidation, ketogenesis, and improved hepatic insulin action in these mice. This indicates that JNK1 and JNK2 in hepatocytes function to reduce glycolysis, fatty acid oxidation, and ketogenesis in response to a high-fat diet [151].

However, in the literature there are also examples where activation of the JNK signaling drives aerobic glycolysis, instead of inhibiting it. Deng et al. [152] reported that JNK1-mediated phosphorylation of Bad (a BH3-only pro-apoptotic Bcl-2 family protein) is required for glycolysis through activation of PFK1. Genetic disruption of *Jnk1* alleles or silencing of *Jnk1* by small interfering RNA abrogates glycolysis induced by growth/survival factors, such as serum or IL-3 [152]. Furthermore, activation of JNK in cortical neurons has been shown to suppress pyruvate metabolism in mitochondria and promote pyruvate conversion to lactate in the cytosol by phosphorylating and inhibiting the PDH complex that normally converts pyruvate to acetyl-CoA, which can then enter into TCA cycle (Fig. 3) [6]. Thus, by inhibiting PDH, the activity of JNK in cortical neurons compromises the oxidative metabolism and favors glycolysis (via lactate production with concomitant NAD+ regeneration) [153]. Of note, like PKM2, PDH is a specific substrate of JNK1 [153]. Activation of the JNK pathway has also been shown to mediate the pro-glycolytic effect of oncogenic RAS expression in primary human keratinocytes. Indeed, keratinocyte-overexpressing RAS mutant exhibited a marked increase in the rate of glycolysis compared to control cells. Such effect was abolished in these cells by treatment with several JNK inhibitors, such as SP600125, indicating that JNK activity plays a central role in RAS-induced glycolysis [154].

In conclusion, the information outlined here indicate that JNK signaling (more likely JNK1) acts as negative regulator of aerobic glycolysis in different types of glycolytic tumors, suggesting an intricate link between JNK and cellular metabolism. These studies also provide evidences for JNK regulating an inextricable crosstalk between apoptosis and cancer metabolism and opens up an interesting opportunity to explore the importance of understanding both the functional roles of each JNK protein in the context of tumor metabolism, in order to validate the therapeutic potential of JNK inhibition in cancer. As pointed out earlier, restraining of the JNK signaling is a common trait of glycolytic tumors [146, 147]. Likewise, survival of many tumors relies on the constitutive activation of the transcription factor NF-κB, which restrains JNK-mediated apoptosis (reviewed in ref. [155]). It would be interesting to understand whether cancers highly dependent on NF-κB activity are more glycolytic than tumors with less active NF-κB. Or whether any of the NF-κB-regulated genes (suppressing JNK signaling) are key regulators of aerobic glycolysis in cancer cells. Notably, NF-κB-mediated restraining of JNK-induced apoptosis is also a mechanistic phenotype activated in response to pro-inflammatory cytokines (i.e., TNF-α) (reviewed in ref. [155]). In this regard, it is imperative querying whether enhanced JNK activation observed in response to TNF-α in NF-κB-deficient cells is associated with inhibition of aerobic glycolysis. Of particular attention are also many examples in the literature in regard to JNK driving aerobic glycolysis. Therefore, future studies aimed at a better understanding of JNK signaling in the regulation of inflammation, cell metabolism, and cancer will likely translate the biology of JNK signaling into a program of drug discovery for inflammatory, metabolic, and cancer diseases.

### Conclusions and future perspectives

A common characteristic of cancer cells that distinguish them from their normal counterparts is an increase in glycolysis with concomitant lactate production (the Warburg effect) [6, 7, 11, 12]. This observation has led to intensive studies of both the molecular mechanisms underlying the Warburg effect and its cellular function with the goal of identifying targeted therapies that are selectively cytotoxic to cancer cells while preserving normal tissue. However, efforts to translate this knowledge into effective therapy is still underway and to date very few drugs targeting the Warburg effect have been approved for clinical evaluation [63, 64]. The main limitations can be surmised as: (1) the metabolic phenotype of cancer cells is also shared by untransformed rapidly proliferating cells, especially cells of the immune system; (2) most glycolytic enzymes are ubiquitously expressed in all mammalian cells and direct targeting of those enzymes could have detrimental side effects. Therefore, targeting oncogenic signaling pathways that affect cellular metabolism may overcome in part limitations of direct targeting of metabolic enzymes. Currently, there are several therapeutic strategies being used to target upstream regulators of metabolic pathways, such as PI3K, AKT, and HIF-1α signaling module [21, 64]. Yet, these targets are also generally shared by normal cells, compromising the development of safe and efficient inhibitors for cancer therapy. Therefore, the identification of pathways that are solely activated in different tumor types is still the best approach to identify drugs against targets that are efficacious and specific for tumors with minimal toxicity on normal cells. In this regard, the BRAF/MEK/ERK pathway represents an ideal candidate for targeting both oncogene and metabolism of certain types of tumors, especially melanomas [98, 106–109], or may work successfully as a combinatorial regimen with other anticancer drugs [109, 129, 130]. Conversely, activating JNK1 that suppresses aerobic glycolysis and favors apoptosis may provide additional therapeutic avenues for glycolytic cancers exhibiting low basal JNK activity, including inflammation-driven cancers (i.e., HCC) and multiple myeloma [135, 155]. In this respect, it is important to note that inhibition of either PARP14 or NF-κB activity may achieve inhibition of tumor metabolism via activation of JNK activity [135, 155]. Further studies into this aspect of cancer cell biology will help to identify targets that will inhibit certain signaling pathways while preserving others and therefore will conceive more efficient antineoplastic agents.
